# Supplementary material for: Single-cell transcriptomics identifies divergent developmental lineage trajectories during human pituitary development
Source: Nat Commun. 2020 Oct 19;11:5275. doi: 10.1038/s41467-020-19012-4 (PMC7572359; doi:10.1038/s41467-020-19012-4)
Supplement: Supplementary file 1 — Supplementary Information [file 41467_2020_19012_MOESM1_ESM.pdf]

Supplementary Information

Zhang et al.: Single-cell Transcriptomics Identifies Divergent Developmental Lineage Trajectories during Human Pituitary Development

Supplementary Fig 1

a

Samles	7W1	8W1	8W2	8W3	9W1	10W1	10W2	13W1	14W1	15W1	16W1	17W1	19W1	19W2	21W1	22W1	22W2	23W1	23W2	24W1	25W1	Sum
Cells#	192	192	240	192	192	144	288	192	240	96	144	288	96	288	96	288	288	96	719	240	670	5181
Filtered#	170	177	214	134	171	129	236	153	179	75	80	262	67	235	77	273	233	61	561	184	442	4113
Endocrine#	95	117	54	120	88	23	161	143	91	32	17	90	56	112	30	225	163	42	306	120	303	2388
Gender	F	M	F	M	M	F	F	M	F	F	F	M	F	F	M	M	F	M	M	F	M	11F, 10M

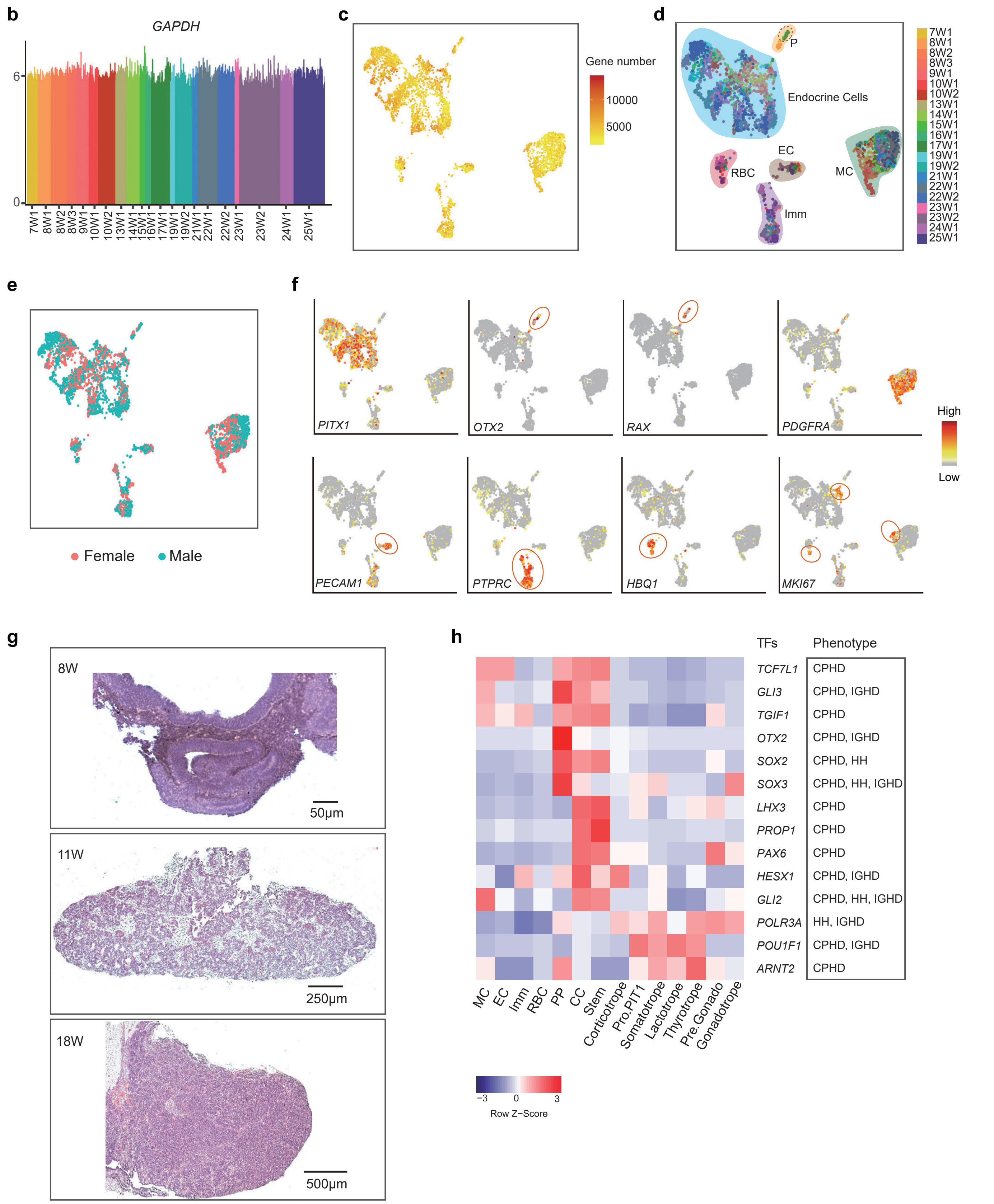

Supplementary Fig 1. Single-cell RNA-seq Information for the Human Fetal Pituitary

(a) Table summarizing sampling information. F, female; M, male.
(b) Bar plot showing the house keeping gene GAPDH in each cell. Color, samples.
(c) Gene number projection on a UMAP plot of all filtered cells (n = 4,113). Deeper red, higher gene number.
(d) Sampling effect surveys on a UMAP plot. Color, sample. Dotted circle: cells collected cells from the posterior pituitaries of fetuses 15W1 and 17W1. P, pituicytes; MC, mesenchymal cells; EC, endothelia cells; Imm, immune cells; RBC, red blood cells.
(e) Distribution of the female and male samples on all pituitary cells shown in the UMAP plot.
(f) Scatterplots showing expression of known markers projected on UMAP plot of all filtered cells. Gray, no expression; deeper red, higher relative expression.
(g) Hematoxylin and eosin (H&E) staining of human fetal pituitary samples from 8-, 11- and 18-week fetuses. In the pituitary of an 8-week fetus, Rathke's pouch was evident, and the anterior pituitary had expanded ventrally; the anterior pituitary eventually expanded to a larger size, as shown in the pituitaries of the 11- and 18-week fetuses.
(h) Heatmap of the averaged z-scored expression (red, high; blue, low) of TFs related pituitary diseases in each cell type. The piuitary phenotypes are shown including combined pituitary hormone deficiency (CPHD), isolated GH deficiency (IGHD) and hypogonadotropic hypogonadism (HH).

Supplementary Fig 2

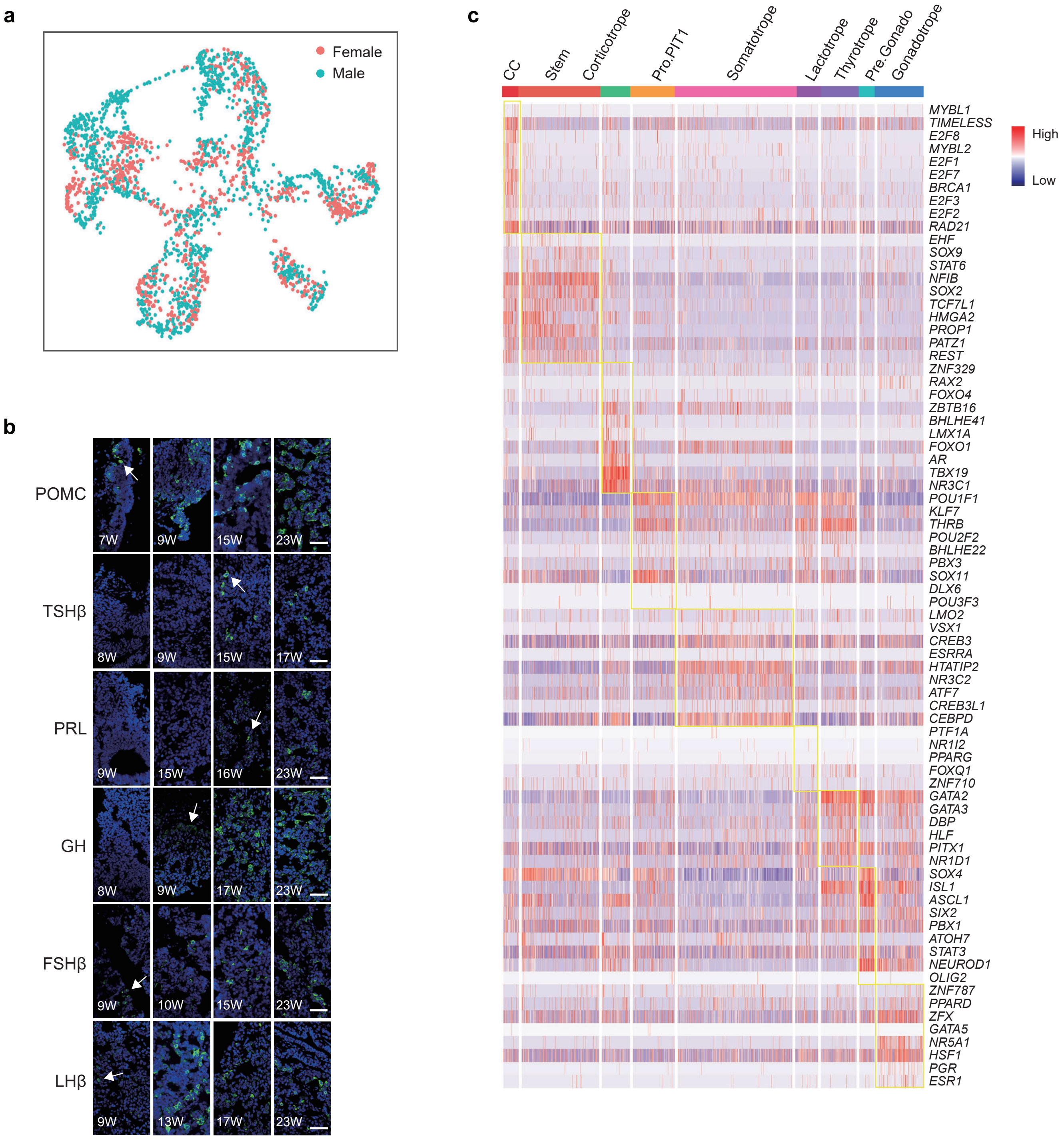

Supplementary Fig 2. Molecular Characteristics of Endocrine Cells
(a) Distribution of the female and male samples on the endocrine cells shown in the UMAP plot.
(b) Time of hormone protein occurrence. Immunofluorescence staining for hormone markers. The first detected stained cells are indicated with white arrows. Scale bar, 50 μm.
(c) Heatmap of the z-scored expression (red, high; blue, low) of the top 10 differentially activated TFs in each cell type corresponding to Fig. 1e.

Supplementary Fig 3

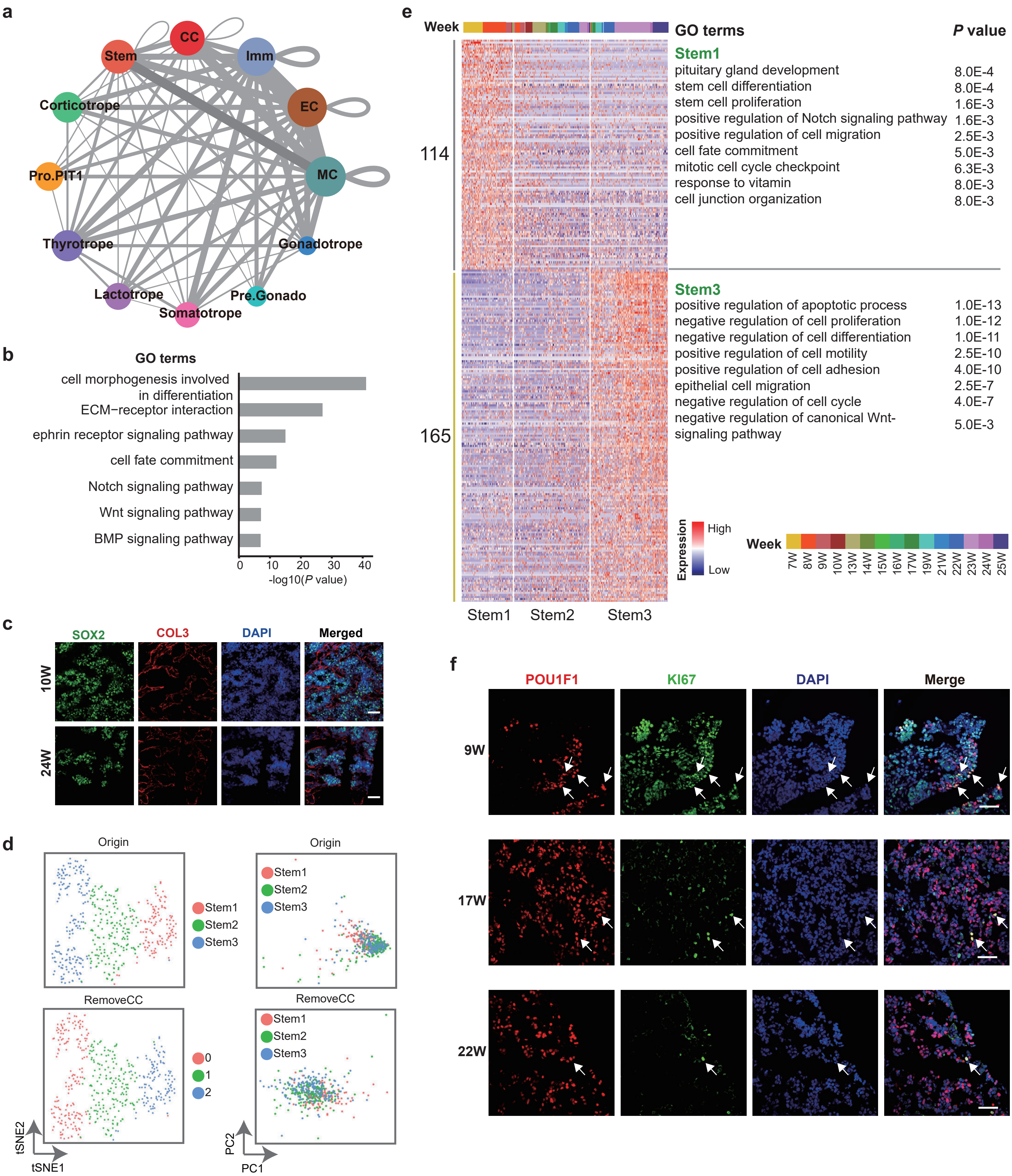

Supplementary Fig 3. Heterogeneity of Stem Cells and Proliferating Cells

(a) Network visualizations of potential specific interactions between each combination of two cell types in the human fetal pituitary. Nodes, cell types; node size, number of all of the significant L-R pairs between specific cell types; edge width, number of significant L-R pairs between each pair of two cell types.

(b) Bar plot showing GO terms of significant paired ligands and receptors in stem cells and mesenchymal cells. P values are determined by one-sided test.

(c) Immunofluorescence staining of SOX2 and COL3 showing that the mesenchymal cells encompass the stem cells. Scale bar, 50 μm.

(d) t-SNE plots (left) showing the subclusters of the stem cells before (upper panels) and after (lower panels) regressing out the cell cycle genes. In addition, the PCA plots (right) using the cell cycle genes (PC1: 43 S phase genes, PC2: 54 G2/M phase genes) could not distinguish between these subclusters.

(e) Differences between subtypes of stem cells. Left, heatmap of the z-scored expression (red, high; blue, low) of DEGs in stem cell subtypes. The numbers of DEGs in Stem1 and Stem3 are shown on the left of the heatmap. Right, GO terms for Stem1 and Stem3. P values are determined by one-sided test.

(f) Immunofluorescence staining for PIT1 and Ki67 in 9-, 17- and 22-week human fetal pituitaries. Arrows, double positive cells. Scale bar, 50μm.

Supplementary Fig 4

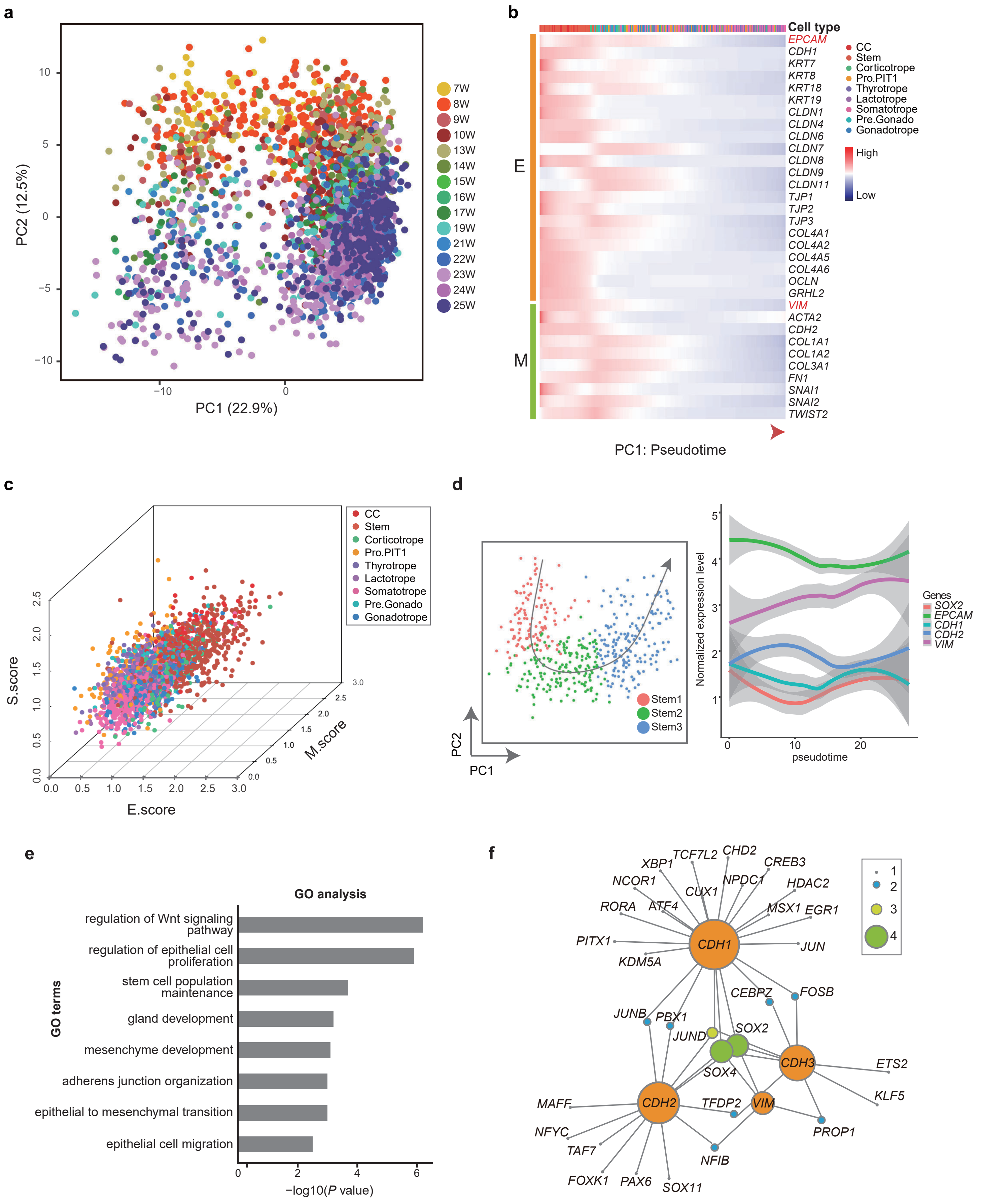

Supplementary Fig 4. Hybrid E/M State of Stem Cells

(a) PCA plot showing differentiation of endocrine cells along the PC1 axis. Dots, single cells; color, week.

(b) Heatmap of the relative expression of representative EMT markers. Colors, loess-smoothed expression (red, high; blue, low). Columns are cells ordered along the PC1 pseudotime axis, and cell types are shown above the heatmap. Rows, genes.

(c) 3D plot of the E.score, M.score, and S.score. Dots, single cells; color, cell type.

(d) Pseudotime analysis by Slingshot projected on PCA plot of stem cells (left) and expression level of EMT markers along the pseudotime of stem cells (right).

(e) Network visualization of predicted TFs binding to the highly expressed EMT markers CDH1, CDH2, CDH3 and VIM. The color and node size indicate the number of directed edges. P values are determined by one-sided test.

(f) GO terms of target genes of PROP1 predicted by the SCENIC workflow.

Supplementary Fig 5

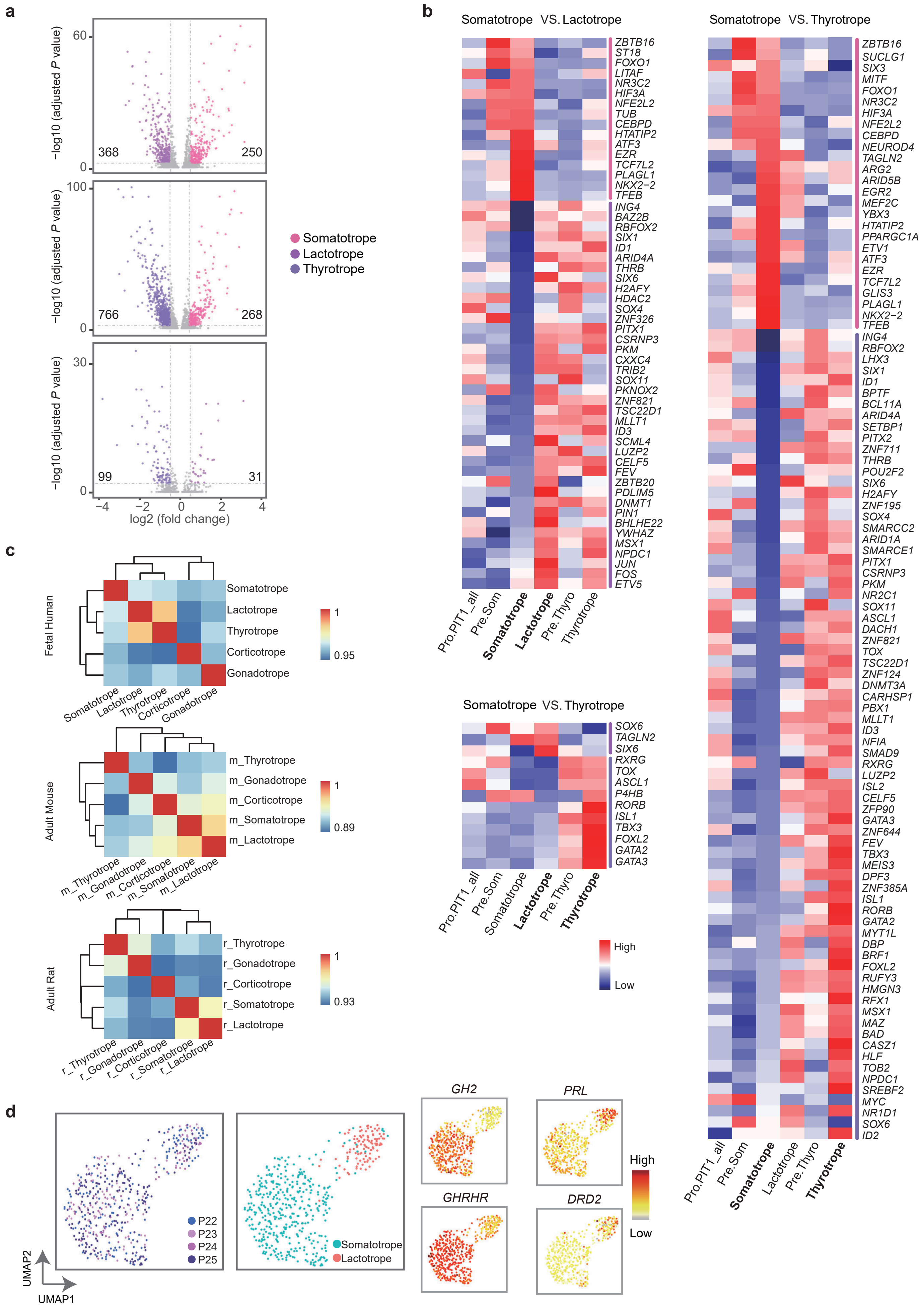

Supplementary Fig 5. Differences among PIT-1 Subtypes and Gene Dynamics of PIT-1 Lineages

(a) Volcano plots showing DEGs between each two HPC of the PIT-1 lineage. Color, DEGs of each cell type. Number of DEGs showed near the corresponding DEGs. P values are determined by two-sided Wilcoxon test and adjusted by Bonferroni correction.

(b) Heatmap of z-scored expression level of differentially expressed TFs between each two HPC of the PIT-1 lineage. Colors, red, high; blue, low. Columns were cells. Rows were genes.

(c) Heatmaps of pearson correlations between the endocrine cell types of human fetal pituitary and rodent adult pituitaries.

(d) Removing batch effect of the somatotrope and lactotrope. Left, cells on the UMAP plot, color, cell type or week; right, gene expression level of representative markers; gray, no expression; deeper red, higher relative expression.

Supplementary Fig 6

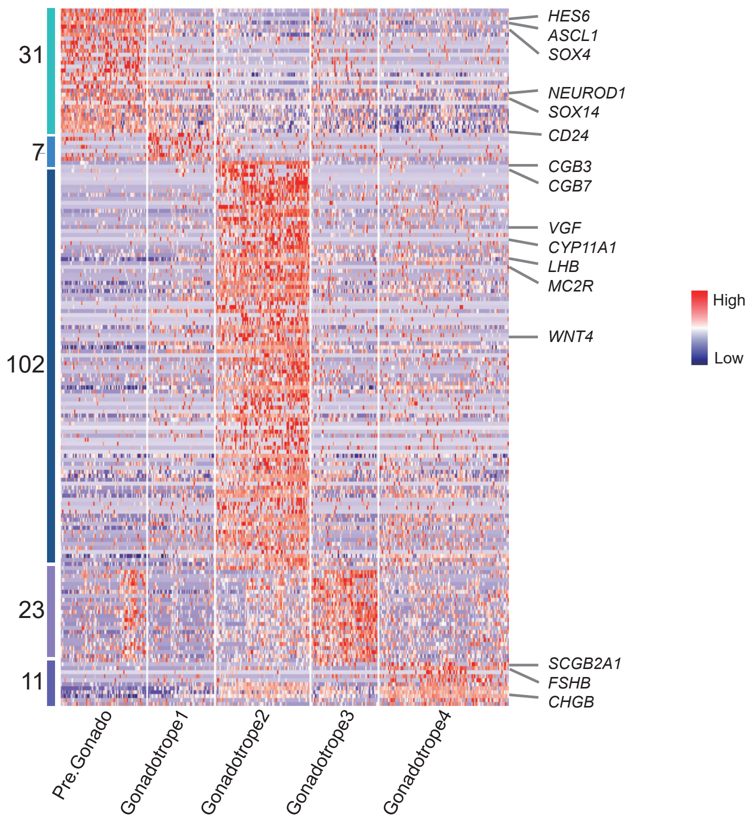

Supplementary Fig 6. Pseudotime Analysis and Gene Dynamics of Gonadotrope Lineages
Heatmap of z-scored expression (red, high; blue, low) of DEGs among gonadotrope subtypes. Column, cells. Rows, genes. Number of DEGs of each subtype are shown on the left of heatmap.

Supplementary Fig 7

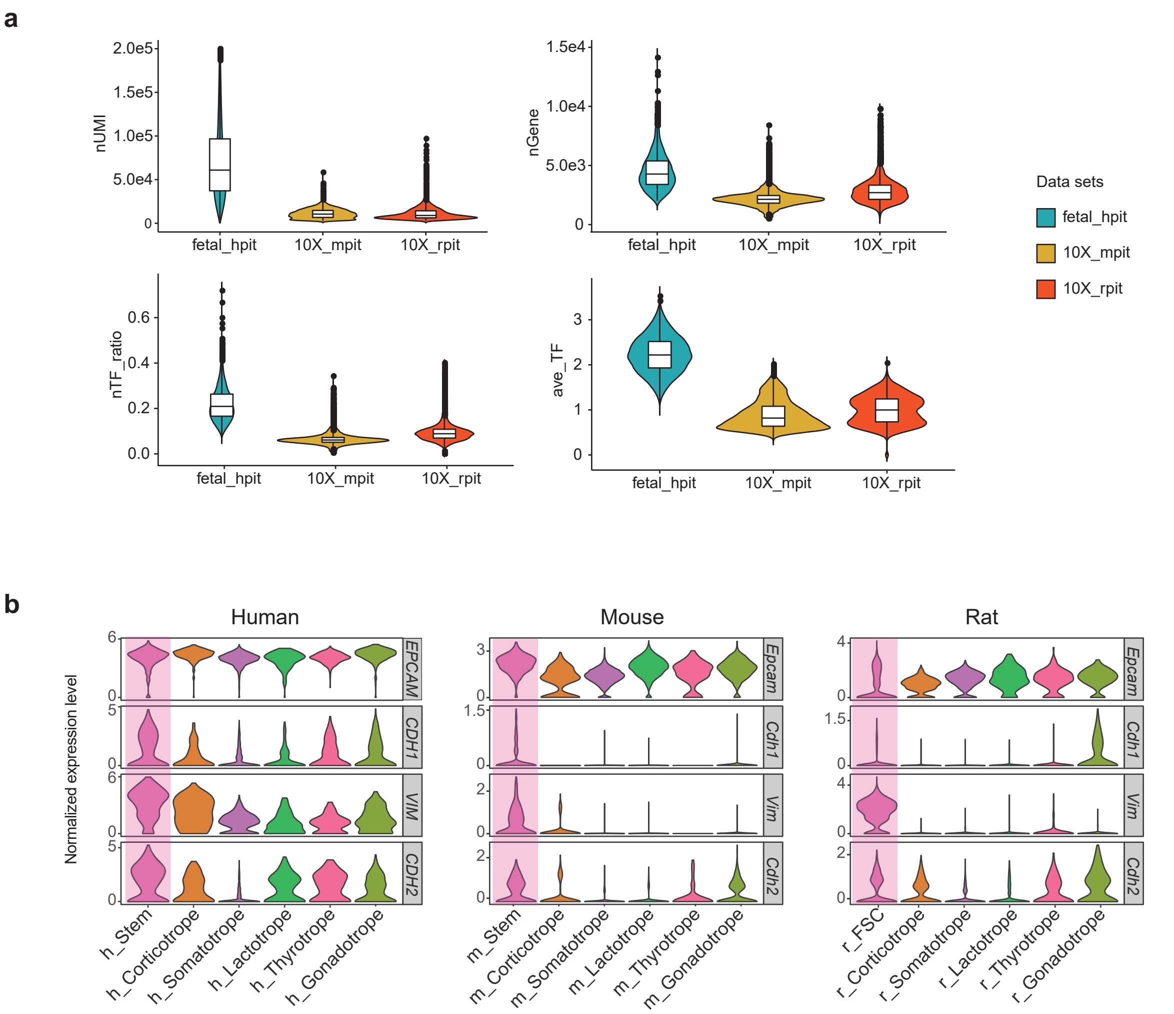

Supplementary Fig 7. Comparison between Human and Rodent Pituitaries.

(a) Comparing the number of genes (nGene) and TFs (nTF_ratio) among human, mouse and rat scRNA-seq datasets.

(b) Violin plots showing the conserved expression of Epcam, Cdh1, Vim and Cdh2 between human and rodent pituitaries.
