# Supplementary material for: Single-cell transcriptomics identifies divergent developmental lineage trajectories during human pituitary development
Source: Nat Commun. 2020 Oct 19;11:5275. doi: 10.1038/s41467-020-19012-4 (PMC7572359; doi:10.1038/s41467-020-19012-4)
Supplement: Supplementary file 10 — Description of Additional Supplementary Files [file 41467_2020_19012_MOESM10_ESM.pdf]

**Title:** Supplementary Data 1:

**Description:** Putative ligands and receptors pairs between the stem and mesenchymal cells.

**Title:** Supplementary Data 2:

**Description:** Genes for defining the epithelial, mesenchymal and stemness scores.

**Title:** Supplementary Data 3:

**Description:** TFs underwent significant changes along the pseudotime axis of each lineage in Fig 4b.

**Title:** Supplementary Data 4:

**Description:** DEGs between Corticotrope1 and Corticotrope2.

**Title:** Supplementary Data 5:

**Description:** DEGs between each two of the somatotrope, the lactotrope and the thyrotrope.

**Title:** Supplementary Data 6:

**Description:** DEGs between Gonadotrope2 and Gonadotrope4.

**Title:** Supplementary Data 7:

**Description:** Cell type-specific DEGs between human and rodent.
