# Supplementary material for: Single-cell transcriptomics identifies divergent developmental lineage trajectories during human pituitary development
Source: Nat Commun. 2020 Oct 19;11:5275. doi: 10.1038/s41467-020-19012-4 (PMC7572359; doi:10.1038/s41467-020-19012-4)
Supplement: Supplementary file 11 — Reporting Summary [file 41467_2020_19012_MOESM11_ESM.pdf]

nature research

Corresponding author(s): Lu Wen

Last updated by author(s): Sep 4, 2020

Reporting Summary

Nature Research wishes to improve the reproducibility of the work that we publish. This form provides structure for consistency and transparency in reporting. For further information on Nature Research policies, see our Editorial Policies and the Editorial Policy Checklist.

Statistics

For all statistical analyses, confirm that the following items are present in the figure legend, table legend, main text, or Methods section.

n/a	Confirmed	
☐	☒	The exact sample size (n) for each experimental group/condition, given as a discrete number and unit of measurement
☐	☒	A statement on whether measurements were taken from distinct samples or whether the same sample was measured repeatedly
☐	☒	The statistical test(s) used AND whether they are one- or two-sided<br>*Only common tests should be described solely by name; describe more complex techniques in the Methods section.*
☐	☒	A description of all covariates tested
☐	☒	A description of any assumptions or corrections, such as tests of normality and adjustment for multiple comparisons
☐	☒	A full description of the statistical parameters including central tendency (e.g. means) or other basic estimates (e.g. regression coefficient) AND variation (e.g. standard deviation) or associated estimates of uncertainty (e.g. confidence intervals)
☐	☒	For null hypothesis testing, the test statistic (e.g. F, t, r) with confidence intervals, effect sizes, degrees of freedom and P value noted<br>*Give P values as exact values whenever suitable.*
☒	☐	For Bayesian analysis, information on the choice of priors and Markov chain Monte Carlo settings
☐	☒	For hierarchical and complex designs, identification of the appropriate level for tests and full reporting of outcomes
☐	☒	Estimates of effect sizes (e.g. Cohen's d, Pearson's r), indicating how they were calculated

Our web collection on statistics for biologists contains articles on many of the points above.

Software and code

Policy information about availability of computer code

Data collection	No special or proprietary software was used.
Data analysis	R version 3.5.3<br>Version of R packages:<br>shiny_1.3.1<br>Seurat_2.3.4<br>slingshot_1.1.0<br>gam_1.16<br>igraph_1.2.5<br>ggpubr_0.1.6<br>VennDiagram_1.6.18<br>org.Hs.eg.db_3.7.0<br>rescue_1.0.1<br><br>Other softwares:<br>cellphonedb v2.0<br>pyscenic version 0.9.1<br>bedtools-2.25.0.tar.gz<br>bowtie2-2.2.3-linux-x86_64.zip<br>bzip2-1.0.6.tar.gz<br>cowplot_0.7.0.tar.gz<br>cufflinks-2.2.1.Linux_x86_64.tar.gz

tabix-0.2.6.tar.bz2
tophat-2.0.12.Linux_x86_64.tar.gz
samtools-0.1.19.tar.bz2
python2.7
Anaconda-2.2.0-Linux-x86_64.sh
HTSeq (__version__ = "0.6.0")
The ImageJ (1.47v) software was used to count cell number and measure areas.
The details were described in Methods section of the manuscript.

For manuscripts utilizing custom algorithms or software that are central to the research but not yet described in published literature, software must be made available to editors and reviewers. We strongly encourage code deposition in a community repository (e.g. GitHub). See the Nature Research guidelines for submitting code & software for further information.

Data

Policy information about availability of data

All manuscripts must include a data availability statement. This statement should provide the following information, where applicable:

- Accession codes, unique identifiers, or web links for publicly available datasets
- A list of figures that have associated raw data
- A description of any restrictions on data availability

The scRNA-seq data have been deposited to the NCBI under accession number GSE142653. Gene expression patterns of the endocrine cells are also available on the shiny webpage: https://tanglab.shinyapps.io/Human_Fetal_Pituitary_Endocrine_Cells/.

Field-specific reporting

Please select the one below that is the best fit for your research. If you are not sure, read the appropriate sections before making your selection.

- [x] Life sciences
- [ ] Behavioural & social sciences
- [ ] Ecological, evolutionary & environmental sciences

For a reference copy of the document with all sections, see nature.com/documents/nr-reporting-summary-flat.pdf

Life sciences study design

All studies must disclose on these points even when the disclosure is negative.

Sample size	Due to the limitations of experimental materials, we collected 21 human fetal pituitaries from fetuses at 7 to 25 week postfertilization, covering the developmental stages from early to late stages. These fetal pituitaries included 11 female fetuses and 10 male fetuses, and two or three fetal pituitaries were collected from different embryos at the same developmental stage (8W, 10W, 19W, 22W and 23W) to exclude the potential batch effect and individual differences. No statistical method were used to determine sample size.
Data exclusions	We exclude the cells that failed quality control and explicitly define all filter criteria in the Methods.
Replication	We used the same single-cell transcriptome methods to perform the sequencing of each library. And the analysis of the data were reliably reproduced. For each representative immunohistochemistry and immunofluorescence assay, we took the nearby 1-2 weeks as biological replicates ($n \geq 2$) due to sampling limitations. The positive cells in different sections ($n \geq 3$) were counted automatically by ImageJ software or manually.
Randomization	The donors in this study were pregnant women who cannot continue pregnancy because of their own diseases (such as cervical insufficiency, inevitable abortion, infection, eclampsia, ect). We collected the human embryonic pituitaries from the fetuses with morphologically normal head and pituitary. In addition, single cells of collected samples were randomly picked with mouth pipette, and library preparation and sequencing was randomized to avoid batch effects.
Blinding	Not applicable since no specific grouping.

Reporting for specific materials, systems and methods

We require information from authors about some types of materials, experimental systems and methods used in many studies. Here, indicate whether each material, system or method listed is relevant to your study. If you are not sure if a list item applies to your research, read the appropriate section before selecting a response.

Materials & experimental systems

n/a	Involved in the study	
☐	☒	Antibodies
☒	☐	Eukaryotic cell lines
☒	☐	Palaeontology and archaeology
☒	☐	Animals and other organisms
☐	☒	Human research participants
☒	☐	Clinical data
☒	☐	Dual use research of concern

Methods

n/a	Involved in the study	
☒	☐	ChIP-seq
☒	☐	Flow cytometry
☒	☐	MRI-based neuroimaging

Antibodies

Antibodies used	For immunofluorescence, commercial primary antibodies (1:50, Mouse anti-Sox2 antibody, sc365823, Santa Cruz Biotechnology; 1:200, Rabbit Anti-MASH1/Achaete-scute homolog 1 (ASCL1) antibody, ab211327; 1:200, Rabbit Anti-KI67 antibody, ab15580, Abcam; 1:75, Goat Anti-POMC antibody, ab32893, Abcam; 1:50, Mouse Anti-AR antibody, sc-7305, Santa Cruz Biotechnology; Mouse Anti-GH antibody, sc-374266, Santa Cruz Biotechnology; 1:200, Rabbit Anti-NKX2-2 antibody, ab191077, Abcam; 1:500, Rabbit Anti-COL3 antibody, ab7778, Abcam; 1:50, Mouse Anti-PIT1 antibody, sc-25258, Santa Cruz Biotechnology; 1:200, Rabbit Anti-TSHβ antibody, ab155958, Abcam; 1:50, Mouse Anti-PRL antibody, sc-46698, Santa Cruz Biotechnology; 1:100, Rabbit Anti-FSHβ antibody, ab180489, Abcam; 1:100, Rabbit Anti-LHβ antibody, ab150416, Abcam).
Validation	All the antibodies were validated for the species (human) and applications (immunofluorescence) by the corresponding manufacturer, which is described in the manufacturer's website. We explored optimal concentration conditions for our experiments, which described in the Methods section of the manuscript (Also see upper Antibody used):<br>For Immunofluorescence (IF), after washing and dehydration, fixed tissue was embedded in Tissue-Tek O.C.T. Compound (#4583, Sakura) and sectioned at a thickness of 10 μm. Then, the sections were washed, permeabilized, blocked and incubated with commercial primary antibodies at 4°C overnight. We used commercial secondary antibodies that were incubated for 2 hours at room temperature. Finally, the sections were counterstained with DAPI in an antifade solution (P36931, Invitrogen) and then mounted.

Human research participants

Policy information about studies involving human research participants

Population characteristics	Human pituitaries of post-fertilization weeks 7-25 week were obtained from the aborted embryos with written informed consent from the donors. A total of 22 embryos (11 females, 11 males) with normal morphology were collected.
Recruitment	Donors for this research were recruited from Peking University Third Hospital. Before giving consent, donors have access to receive proper counseling about the implications of the donation and potential risks. Embryos with morphologically normal head and pituitary were collected with written informed consent from the donors in this study.
Ethics oversight	The Reproductive Medicine Ethics Committee of Peking University Third Hospital

Note that full information on the approval of the study protocol must also be provided in the manuscript.
